# Control of foliar pathogens of spring barley using a combination of resistance elicitors

**DOI:** 10.3389/fpls.2014.00241

**Published:** 2014-05-28

**Authors:** Dale R. Walters, Neil D. Havis, Linda Paterson, Jeanette Taylor, David J. Walsh, Cecile Sablou

**Affiliations:** ^1^Crop and Soil Systems Research Group, Scotland's Rural CollegeEdinburgh, UK; ^2^Engineering, Science and Technology Department, Scotland's Rural CollegeEdinburgh, UK

**Keywords:** *Blumeria graminis* f.sp. *hordei*, disease control, elicitors, induced resistance, *Rhynchosporium commune*, systemic acquired resistance

## Abstract

The ability of the resistance elicitors acibenzolar-S-methyl (ASM), β-aminobutyric acid (BABA), *cis*-jasmone (CJ), and a combination of the three products, to control infection of spring barley by *Rhynchosporium commune* was examined under glasshouse conditions. Significant control of *R. commune* was provided by ASM and CJ, but the largest reduction in infection was obtained with the combination of the three elicitors. This elicitor combination was found to up-regulate the expression of *PR-1b*, which is used as a molecular marker for systemic acquired resistance (SAR). However, the elicitor combination also down-regulated the expression of *LOX2*, a gene involved in the biosynthesis of jasmonic acid (JA). In field experiments over 3 consecutive years, the effects of the elicitor combination were influenced greatly by crop variety and by year. For example, the elicitor combination applied on its own provided significant control of powdery mildew (*Blumeria graminis* f.sp. *hordei*) and *R. commune* in 2009, whereas no control on either variety was observed in 2007. In contrast, treatments involving both the elicitor combination and fungicides provided disease control and yield increases which were equal to, and in some cases better than that provided by the best fungicide-only treatment. The prospects for the use of elicitor plus fungicide treatments to control foliar pathogens of spring barley in practice are discussed.

## Introduction

Application of various agents to plants can lead to the induction of resistance to subsequent pathogen attack, both locally, and systemically (Walters et al., 2013). Such induced resistance can be split into systemic acquired resistance (SAR) and induced systemic resistance (ISR). SAR is characterized by a restriction of pathogen growth and a suppression of disease symptom development compared to non-induced plants infected with the same pathogen. Its onset is associated with an accumulation of salicylic acid (SA) at sites of infection and systemically, and with the coordinated activation of a specific set of genes encoding pathogenesis-related (PR) proteins. Treatment of plants with SA or one of its functional analogs e.g., acibenzolar-S-methyl (ASM; marketed in Europe as Bion® and in North America as Actigard®), induces SAR and activates the same set of *PR* genes. ISR develops as a result of colonization of plant roots by plant growth-promoting rhizobacteria (PGPR) and has been shown to function independently of SA and activation of *PR* genes, requiring instead, jasmonic acid (JA), and ethylene (ET) (Pieterse et al., 2012; Spoel and Dong, 2012).

Research over the past decade suggests that SA induces defenses against biotrophic pathogens, while JA mediates defenses against necrotrophic pathogens (Glazebrook, 2005). It is thought that cross-talk between the two signaling pathways might help to fine-tune defense responses against a particular pathogen according to its mode of infection (Beckers and Spoel, 2006). Interestingly, Spoel et al. (2007) found that infection with the biotrophic pathogen *Pseudomonas syringae*, which induces SA-mediated defense, rendered *Arabidopsis thaliana* more susceptible to the necrotrophic pathogen *Alternaria brassicicola*, although this trade-off was restricted to plant tissue adjacent to the initial infection site. In terms of plant-insect interactions, JA is known to play a major role in mediating defenses against insect herbivory (Bostock, 2005), although the situation is rather more complex than was once thought, since both SA- and JA-responsive gene expression can be elicited by aphids and whiteflies. In terms of insect herbivory, although there are examples of negative cross-talk i.e., SA-mediated suppression of JA-inducible gene expression, there are also examples of no trade-offs, and even of positive effects (see Walters et al., 2013).

Because induced resistance offers the prospect of broad spectrum disease control using the plant's own resistance mechanisms, there has been great interest in the development of agents which can mimic natural inducers of resistance (Lyon, 2007). These include elicitor molecules released during the early stages of the plant-pathogen interaction and the signaling pathways used to trigger defenses locally and systemically. Examples include ASM, which has been shown to elicit SAR in a wide range of plant-pathogen interactions (Leadbeater and Staub, 2007), the non-protein amino acid β-aminobutyric acid (BABA), and the oxylipin, *cis*-jasmone (CJ) (Walters et al., 2007).

BABA is known to induce resistance against pathogens in various systems, including tomato, potato, grapevine, and pea (Cohen et al., 1999; Jakab et al., 2001). In field experiments, Cohen (2002) found that BABA provided significant control of late blight of potato, while Liljeroth et al. (2010) showed that BABA used together with a reduced fungicide dose gave the same level of late blight control as a full dose of the standard fungicide treatment.

CJ is structurally related to JA and methyl jasmonate (MeJA), both of which are well known to activate plant defenses (Farmer and Ryan, 1990; Thaler et al., 1996), although CJ activates a unique and more limited set of genes than does treatment with MeJA (e.g., Pickett et al., 2007). CJ is released naturally from plants damaged by insects and when applied artificially, can activate defense against insects (Birkett et al., 2000; Bruce et al., 2003).

The efficacy of induced resistance under field conditions is variable, representing a major obstacle to its use in practical crop protection (Reglinski and Walters, 2009). Induced resistance is a complex plant response to pathogen attack and as such, will be modified by many factors including genotype. However, insufficient attention has been paid to investigating the mechanisms underlying variable efficacy and approaches that might be adopted to incorporate elicitors into crop protection practice, such as use of elicitors and fungicides together in the same disease control program, and use of combinations of elicitors. The latter aspect has received little attention to date, probably because of the trade-offs that might be associated with using elicitors which activate different signaling pathways, as mentioned above. In this paper, we report the results of field experiments over 3 consecutive years, undertaken to determine the potential for use of an elicitor combination to control foliar pathogens of spring barley. Some preliminary data from this study have appeared previously in a conference paper (Walters et al., 2010).

## Materials and methods

### Plant growth and pathogen inoculation under glasshouse conditions

The spring barley (*Hordeum vulgare* L.) variety Cellar was used for glasshouse studies. Cellar was chosen since it exhibits moderate susceptibility to *Rhynchosporium commune* (HGCA, 2009). Seeds were sown in pots in Fisons Levington compost and grown in a walk-in growth room at 18°C with a 16 h photoperiod (190 μmol m^−2^ s^−1^ provided by 400 W mercury vapor lamps). The experiment was laid out in a randomized block design, with each of the five treatment groups consisting of 15 plants, with 10 plants used for disease assessment and 5 plants for gene expression analysis. Plants were used for efficacy experiments when the sixth leaf was fully formed and the seventh leaf emerging. Leaves 1–4 were sprayed with elicitors using a hand-held sprayer. Two days later, plants were inoculated with the leaf scald pathogen, *Rhynchosporium commune*, by spraying with a suspension of spores (1 × 10^5^ spores/ml) in distilled water containing 0.01% Tween 20. Inoculated plants were then covered with plastic bags for 48 h (the first 24 h in the dark) and kept at 16°C to provide the conditions necessary for spore germination and early fungal development. Thereafter, the temperature of the growth room was increased to 18°C for the remainder of the experiment. Infection intensity on leaves 5–7 was assessed 21 days after inoculation by determining the % leaf area exhibiting symptoms on each of 10 plants. For gene expression experiments, leaves three and four were treated with elicitor and 2 days later were inoculated with *R. commune*. Leaves were harvested 2 days later and frozen at −80°C for gene expression analysis. Data presented are the means of three replicates.

The elicitors used in these experiments were ASM, BABA, and CJ. ASM (Bion®) was a gift from Syngenta, Basel, Switzerland; BABA was purchased from Sigma, Poole, Dorset, UK; CJ was purchased from Sigma Aldrich, Dorset, UK. ASM (1 mM), BABA (1 mM) and CJ (0.625 g/l) were made up in distilled water containing 0.01% Tween 20.

### Field experiments

Field experiments were conducted in 2007 at Tibbermore, Perthshire, Scotland, and in 2008 and 2009 at Drumalbin, Lanark, Scotland. Total rainfall and average air temperatures at these sites during the period 1 June—1 September were:

Perthshire, 2007: rainfall = 219 mm; average air temperature = 17°C

Lanark, 2008: rainfall = 317 mm; average air temperature = 16°C

Lanark, 2009: rainfall = 338 mm; average air temperature = 17°C.

Two spring barley varieties (Cellar and Optic) used in all field experiments reported here. Cellar has a resistance rating (RR) of 9 for powdery mildew and 4 for *Rhynchosporium commune*, and Optic has a RR of 5 for powdery mildew and 4 for *R. commune* (RR scale: 10 = high resistance, 1 = low resistance; HGCA, 2009). Each variety was sown in a randomized block design at a seed rate of 360 seeds m^−2^ and an individual plot size of 10 × 2 m, using three plots per treatment. For each barley variety, the factor tested was the applied treatment (i.e., elicitor, fungicide, elicitor + fungicide). Plots received standard fertilizer and herbicide regimes and 16 treatment programs were compared (Table 1). Spray dates for treatments were based on plant growth stage as described by Zadoks et al. (1974) and were applied with a knapsack sprayer using an equivalent spray volume of 200 l ha-1. Disease symptoms (% leaf area infected) and % GLA were assessed using 10 plants per plot at spray dates and at 14 day intervals after the final spray. Area under the disease progress curves (AUDPC) were calculated using the formula:

$$\Sigma \left({\text{y}}_{\text{i}}+\;{\text{y}}_{\left(\text{i}\;+\;1\right)}\right)/2\times \left({\text{t}}_{\left(\text{i}\;+\;1\right)}-{\text{t}}_{\text{i}}\right)$$

where y_i_ is the disease rating at time t_i_.

**Table 1:** **Elicitor and fungicide treatments applied in field experiments in 2007–2009**.

**Treatment**	**GS24**	**GS31**	**GS39**
1	Nil	Nil	Nil
2	Elicitors	Nil	Nil
3	Nil	Elicitors	Nil
4	Nil	Nil	Elicitors
5	Elicitors	Elicitors	Nil
6	Elicitors	Nil	Elicitors
7	Fungicide^1^	Nil	Nil
8	Nil	Fungicide^1^	Nil
9	Nil	Nil	Fungicide^2^
10	Nil	Fungicide^1^	Fungicide^2^
11	Fungicide^1^ + Elicitors	Nil	Nil
12	Nil	Fungicide^1^ + Elicitors	Nil
13	Nil	Nil	Fungicide^2^ + Elicitors
14	Elicitors	Fungicide^1^	Fungicide^2^
15	Elicitors	Fungicide^3^	Fungicide^4^
16	Elicitors	Elicitors + Fungicide^3^	Fungicide^4^

*Treatments were applied at different growth stages (GS) as described in the Materials and Methods*.

*GS = growth stage (see Zadoks et al., 1974)*.

*Elicitors = ASM + BABA + C*.

*Fungicide^1^ = prothioconazole at full-rate x 0.4 + cyprodinil and picoxystyrobin at full-rate x 0.5*.

*Fungicide^2^ = prothioconazole at full-rate x 0.4 + chlorothalonil at full-rate*.

*Fungicide^3^ = prothioconazole at full-rate x 0.2 + cyprodinil and picoxystrobin at full-rate x 0.3*.

*Fungicide^4^ = prothioconazole at full-rate x 0.2 + chlorothalonil at full-rate x 0.5*.

Plots were harvested at the end of the trial and yields expressed as tonnes/hectare at 85% dry matter content.

### Gene expression

Total RNA was extracted from barley leaves using a RNeasy™ kit (Qiagen, West Sussex, UK) and RNA yield determined using a Nanodrop 1000 spectrophotometer (Nanodrop Technologies, Wilmington, DE, USA). In order to remove any remaining trace of DNA likely to interfere with measurements, samples were treated with desoxyribonuclease enzymes using the DNA-*free*™ kit from Applied Biosystems (California, USA). The final quantity and quality of the RNA was tested using a RNA 6000 Nano Chip kit (Agilent Technologies, Santa Clara, CA, USA).

Primer sequences for *PR1-b, LOX2*, and the cyclophilin gene (internal control) are listed in Table 2. All sequences were purchased from Eurofins MWG Operon (Ebersburg, Germany) and all primers were designed using Beacon Designer software (Premier Biosoft International, Palo Alto, California, USA).

**Table 2:** **Primer sequences for genes used in this study**.

**Gene**	**Primer sequence (F)**	**Primer sequence (R)**
*PR1-b*	CTACGACTACGGCTCCAACAC	GCATCACGGTTAGTATGGTTTCTG
*LOX2*	CGGCAGACTCCCTCATCACTAAAG	GGCAGCAACAGGTCGTGGTAG
*Cyclophilin*	CCTGTCGTGTCGTCGGTCTAAA	ACGCAGATCCAGCAGCCTAAAG

*Amplicon lengths: PR1-b = 190 base pairs. LOX2 = 121 base pairs. Cyclophilin = 122 base pairs*.

Following RNA extraction, cDNA was generated using a SuperScript™ first-strand cDNA synthesis kit (Invitrogen, USA). Quantitative real-time PCR (qRT-PCR) was then performed with a MX3000P system (Stratagene, CA, USA) using a Brilliant 11SYBR Green QPCR master mix with ROX (Agilent Technologies, Santa Clara, CA, USA). In order to construct standard curves for the genes, six data points were used with a 5-fold dilution series (1:10–1:31,250). A 25 μl reaction for PCR amplification contained 12.5 μl of SYBR Green master mix (see above), 0.75 μl forward primer, 0.75 μl reverse primer, 6 μl sterile distilled water, and 5 μl cDNA. All PCR reactions were performed in duplicate. The cycling conditions were as follows: pre-incubation for 10 min at 95°C, followed by 40 cycles, each consisting of 30 s denaturing at 95°C, 60 s annealing at 57°C, 30 s at 72°C for new strand synthesis. The standard curves were used to calculate the absolute quantity of the product in each sample, Relative expression values were then calculated by normalizing against the cyclophilin gene as an internal control.

### Statistical analysis

All data were subjected to One-Way ANOVA using the GenStat Release 11.1 statistical program. The effect of blocks was considered random and the effect of applied treatments was defined as fixed. % leaf area infected values from glasshouse experiments and % GLA data from field experiments were log-transformed prior to analysis. Comparison of treatment means was performed using Fisher's protected least significant difference (LSD) Test.

## Results

### Effects of elicitors under glasshouse conditions

**Figure 1:**
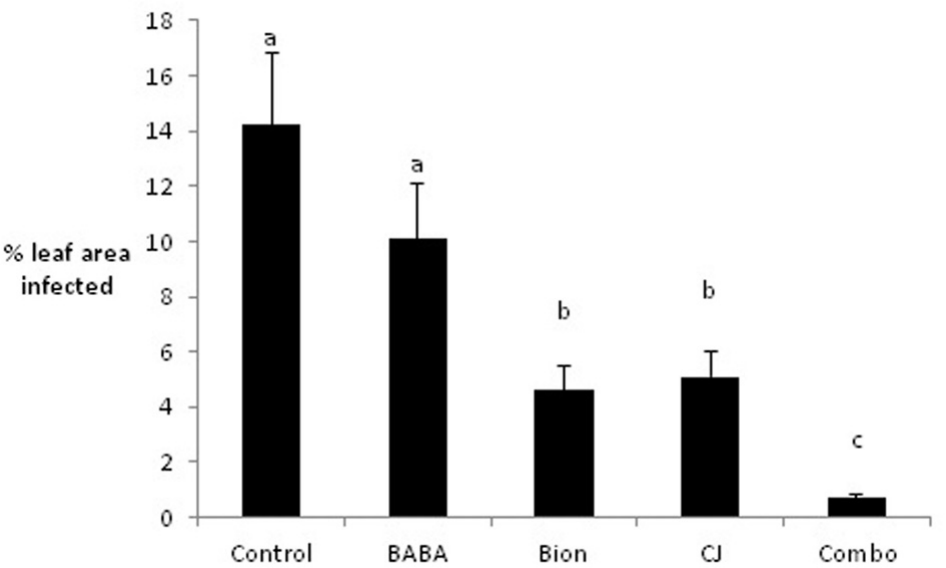
**Effects of elicitors, singly and in combination, on infection of barley with *R. commune***. Leaves 1–4 were sprayed with elicitor and inoculated with *R. commune* 2 days later. Infection intensity was assessed 21 days later on leaves 5–7. Bars with a different letter are significantly different at *P* < 0.05 (Fisher's LSD).

Initial experiments were conducted under glasshouse conditions to examine the effects of Bion®, BABA, and CJ, singly and in combination, on infection of the barley variety Cellar by *R. commune*. Although BABA reduced infection compared to the untreated control, this difference was not significant. In contrast, treatment with Bion® and CJ led to significant reductions in *R. commune* infection, with Bion® reducing infection by 70% and CJ by 64% (Figure 1). The largest reduction in infection (96%) was obtained using a combination of Bion®, BABA, and CJ.

**Figure 2:**
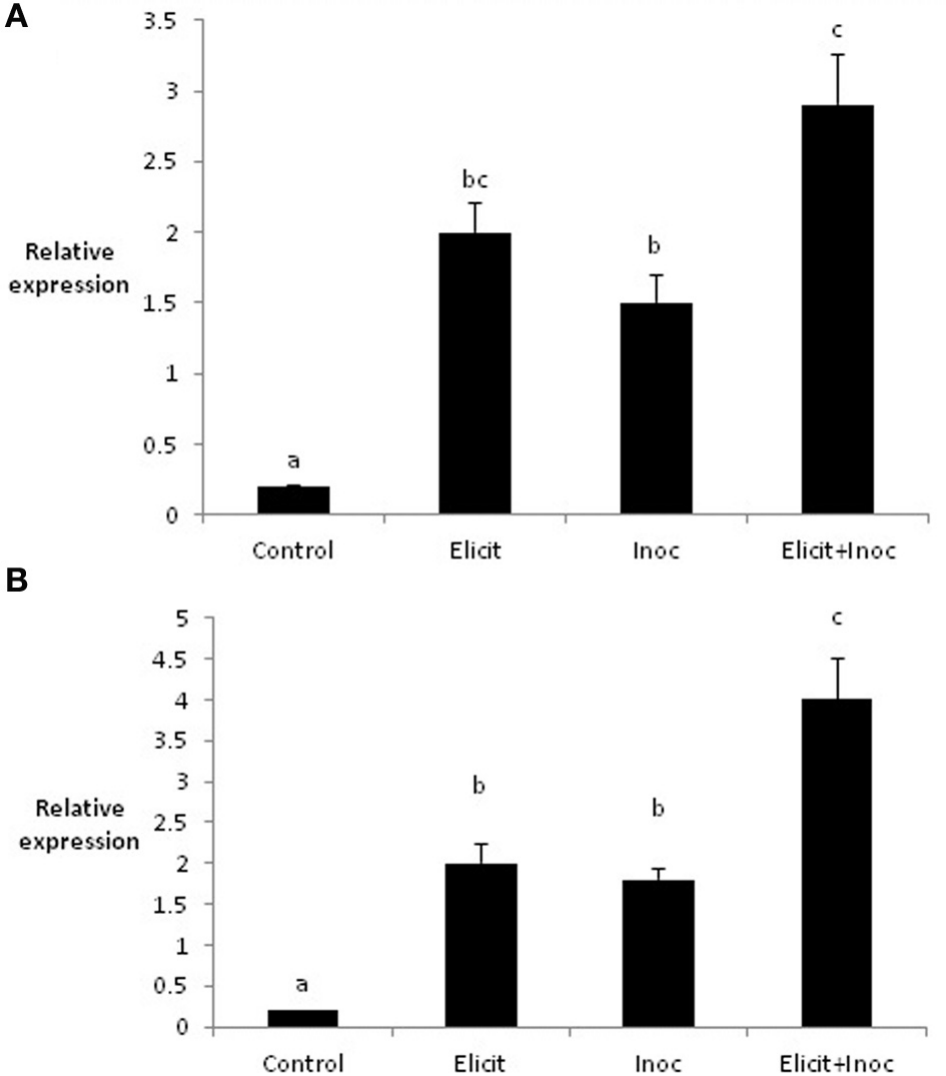
**Relative quantity of *PR1-b* in leaf 3 (A) and leaf 4 (B) of barley plants treated with the elicitor combination**. Leaves three and four were treated with elicitor and inoculated with *R. commune* 2 days later. Leaves were harvested 2 days after inoculation for analysis of gene expression. Bars with a different letter are significantly different at *P* < 0.05 (Fisher's LSD).

Application of the elicitor combination to leaves 1 and 2 of the barley variety Cellar led to changes in the expression of two defense-related genes in leaves 3 and 4. Thus, elicitor treatment resulted in significant increases in expression of *PR1b* in both leaves 3 and 4 (4.3-fold and 3.8-fold, respectively; Figures 2A,B). Expression of *PR1b* was also increased significantly in leaf 3 following inoculation of untreated leaves (2.6-fold increase), but was not affected in leaf 4 (Figures 2A,B). However, the largest increases in *PR1b* expression were obtained when leaves 3 and 4 were first treated with elicitor and subsequently inoculated with *R. commune*. Here, *PR1b* expression was increased 7-fold in leaves 3 and 4 compared to the untreated control (Figures 2A,B). This suggests that the elicitor combination primes the plant for enhanced expression of *PR1b*.

**Figure 3:**
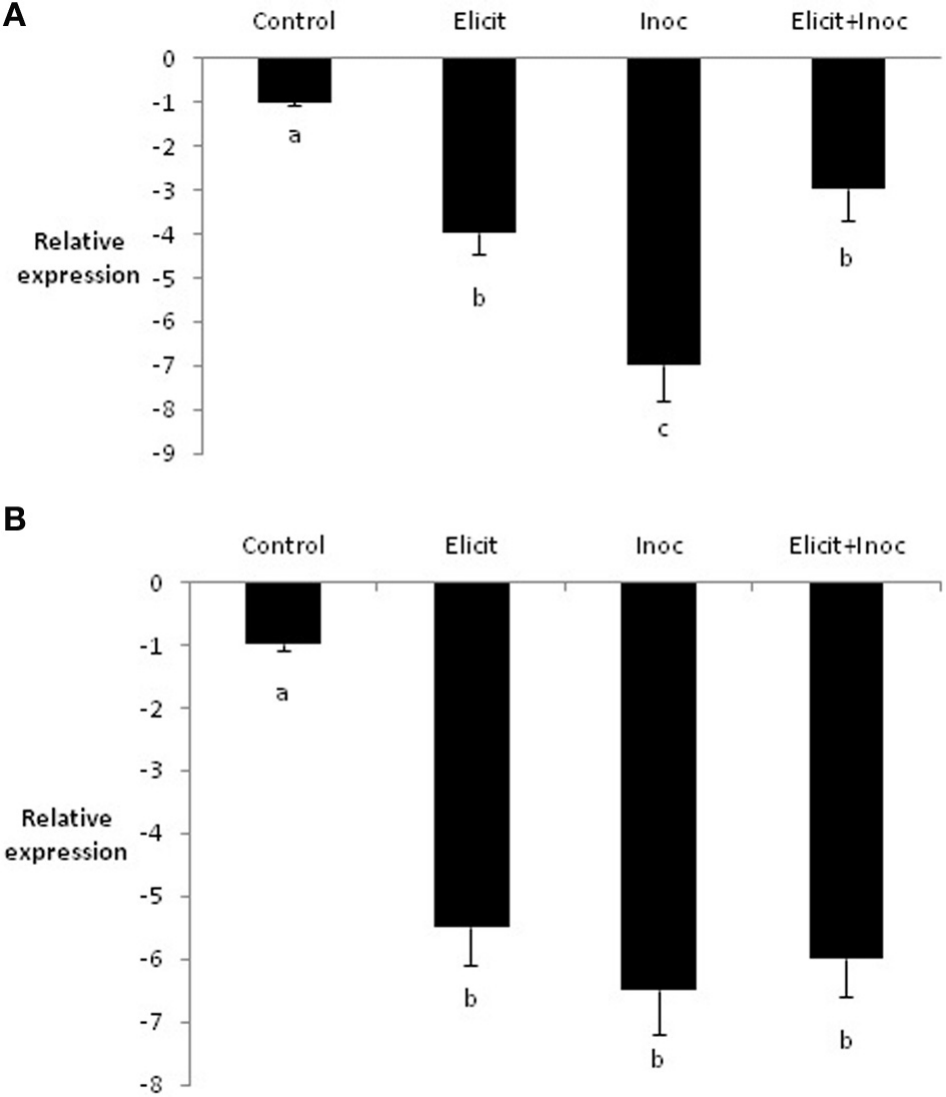
**Relative quantity of *LOX2* in leaf 3 (A) and leaf 4 (B) of barley plants treated with the elicitor combination**. Leaves three and four were treated with elicitor and inoculated with *R. commune* 2 days later. Leaves were harvested 2 days after inoculation for analysis of gene expression. Bars with a different letter are significantly different at *P* < 0.05 (Fisher's LSD).

In contrast to *PR1b*, expression of *LOX2* was reduced by treatment with the elicitor combination, compared to the untreated control. Indeed, all three treatments led to substantial and significant decreases in expression of *LOX2* in leaves 3 and 4 (Figures 3A,B).

### Field experiments

Since the elicitor combination provided most effective control of *R. commune* under glasshouse conditions, this treatment was chosen for inclusion in field experiments. Over the 3 years of field experiments, the two major foliar diseases detected on the spring barley crops were powdery mildew and *R. commune*. However, disease levels varied between years and in some years (2008 and 2009), only *R. commune* was observed on the variety Cellar. This probably reflected differences in weather at the different sites, since, for example, the 2007 season at the Perth site was considerably drier than the Lanark sites in 2008 and 2009.

The 3 years of field experimentation, involving 17 different treatments on two varieties generated too much data to be shown here. Instead, only data for selected treatments are shown. These treatments are untreated (1), one fungicide-only treatment (10), one elicitor-only treatment (5), and one elicitor + fungicide (reduced rate) treatment (13). The data for all treatments are provided in Supplementary Material.

**Figure 4:**
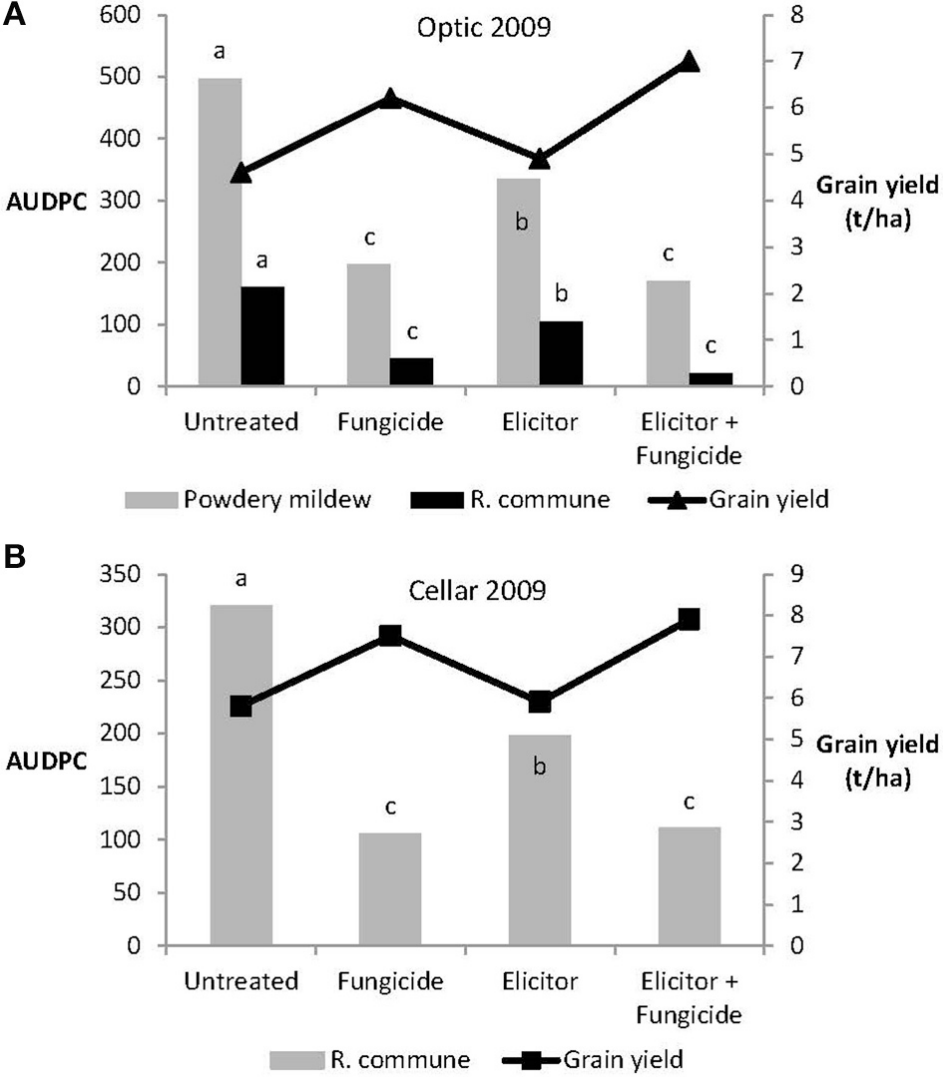
**Effects of the elicitor combination and fungicides on (A) AUDPC for powdery mildew and *R. commune*, and grain yield in the spring barley variety Optic, and (B) on AUDPC for *R. commune* and grain yield in the variety Cellar, in a field experiment in 2009**. Bars with different letters are significantly different at *P* < 0.05 (Fisher's LSD).

The efficacy of the elicitor combination was dependent on crop variety and year. Thus, in 2009, the elicitor combination provided significant control of both powdery mildew and *R. commune* on the variety Optic and of *R. commune* on variety Cellar (Figure 4). However, on both varieties, most effective disease control was achieved using a combination of the elicitor combination and fungicide. Indeed, the level of disease control achieved with the elicitor and fungicide treatment was at least as good as that obtained using the fungicide only treatment (Figure 4). Interestingly, although the elicitor combination on its own provided significant disease control on the two varieties, grain yield remained unchanged compared to untreated plants (Figure 4). In contrast, grain yield was increased significantly (*P* < 0.05) in both barley varieties treated with the elicitor plus fungicide treatment (Figure 4).

**Figure 5:**
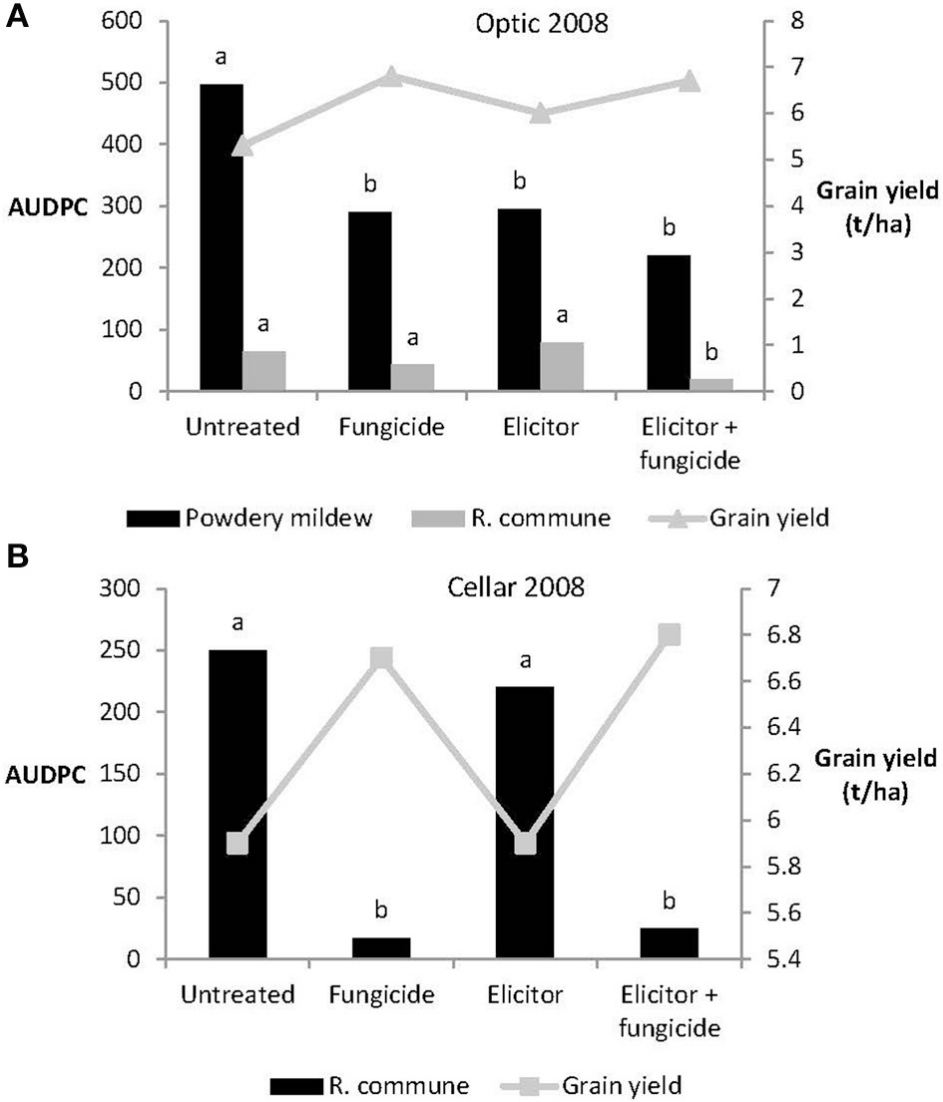
**Effects of the elicitor combination and fungicides on (A) AUDPC for powdery mildew and *R. commune*, and grain yield in the spring barley variety Optic, and (B) on AUDPC for *R. commune* and grain yield in the variety Cellar, in a field experiment in 2008**. Bars with different letters are significantly different at *P* < 0.05 (Fisher's LSD).

In 2008, the elicitor combination applied on its own reduced levels of powdery mildew significantly on variety Optic, but provided no control of *R. commune* on either Optic or Cellar (Figure 5). Treatment with elicitor plus fungicide provided significant control of both diseases on Optic and of *R. commune* on Cellar. In both varieties, highest grain yields were obtained from plants receiving the elicitor plus fungicide treatment (Figure 5).

**Figure 6:**
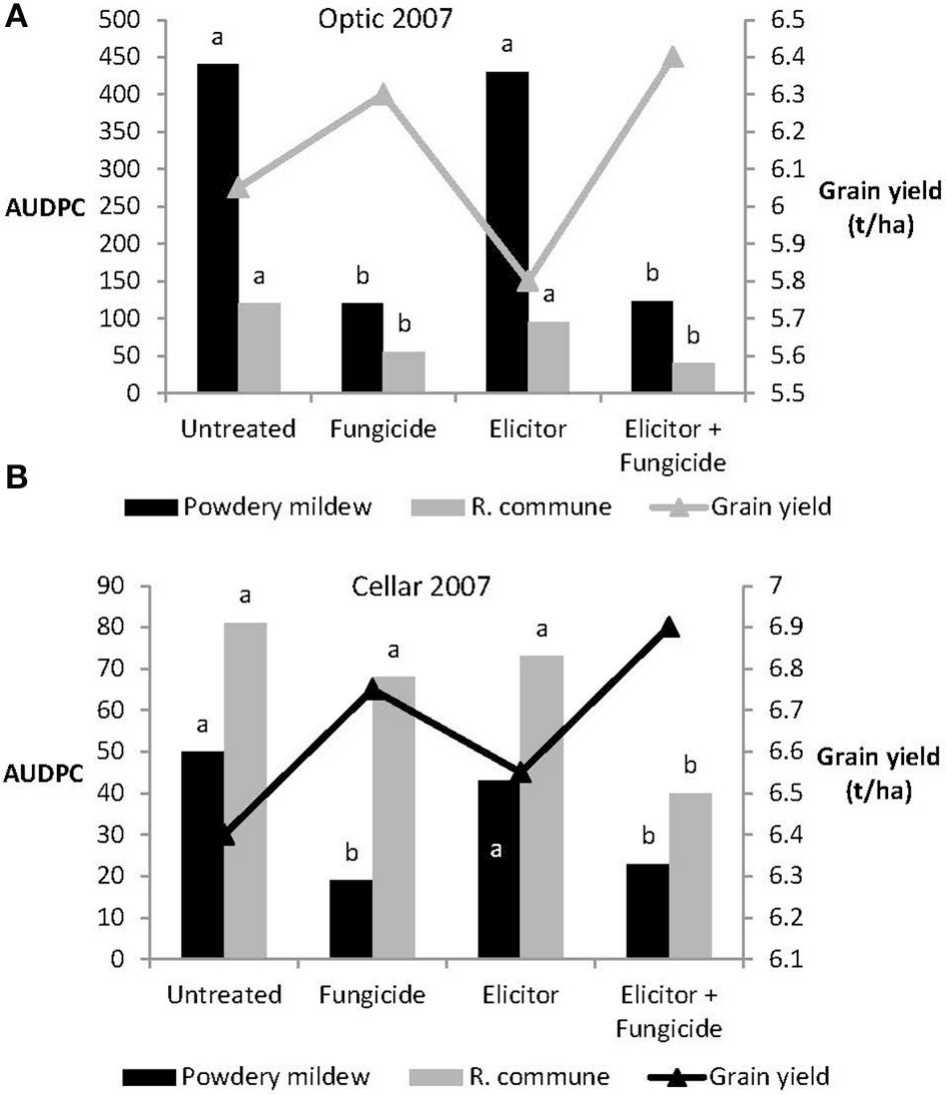
**Effects of the elicitor combination and fungicides on (A) AUDPC for powdery mildew and *R. commune*, and grain yield in the spring barley variety Optic, and (B) in the variety Cellar, in a field experiment in 2007**. Bars with different letters are significantly different at *P* < 0.05 (Fisher's LSD).

Levels of both powdery mildew and *R. commune* were not significantly affected by treatment of either variety with the elicitor combination on its own in 2007 (Figure 6). In contrast, on both varieties, treatment with elicitor plus fungicide provided significant control of both diseases and in most cases the level of disease control achieved was as good as that obtained using the fungicide only treatment (Figure 6). Grain yields of both varieties were significantly increased in plants receiving the elicitor plus fungicide treatment compared to the other treatments, while in Optic, the elicitor combination on its own actually reduced grain yield (Figure 6).

**Figure 7:**
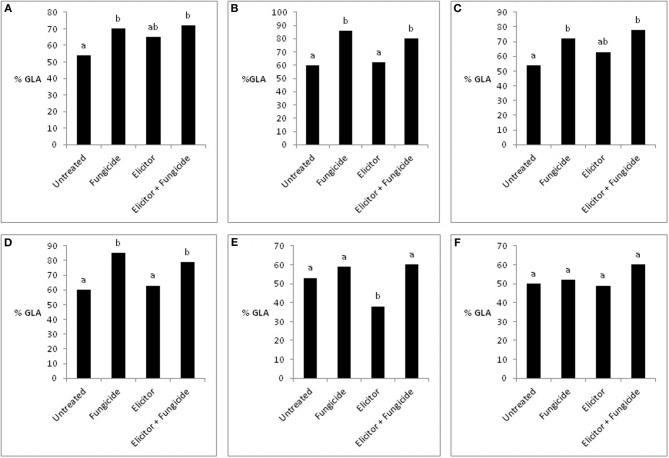
**Effects of the elicitor combination and fungicides on percentage green leaf area (% GLA) in Optic and Cellar in 2009 (A,B), Optic and Cellar in 2008 (C,D), and Optic and Cellar in 2007 (E,F)**. Bars with different letters are significantly different at *P* < 0.05 (Fisher's LSD).

The absence of any significant effect of the elicitor-only treatment on grain yields in the 2 varieties across the years probably reflects, in part, the lack of a significant effect of this treatment on green leaf area (GLA) (Figure 7). This is supported by the fact that the significant reduction in grain yield in the variety Optic in 2007 (Figure 6) was associated with a significant reduction in GLA (Figure 7E). This contrasts with the elicitor plus fungicide treatment, where, in most cases, increased grain yields (Figures 4–6) were associated with significantly increased GLAs (Figure 7). The only exception was the variety Cellar in 2007, where increased grain yield in the elicitor plus fungicide treatment (Figure 6B) was accompanied by a small, but statistically non-significant increase in GLA (Figure 7F).

## Discussion

Under glasshouse conditions, ASM and CJ reduced *R. commune* infection of barley by 64–70%, while BABA had much less effect. Although there are many examples of disease control provided by ASM and BABA (Cohen et al., 2010; Walters et al., 2013), to our knowledge, this is the first report of disease control provided by CJ. Interestingly, applying the three elicitors together provided the best levels of disease control, reducing infection by 96%, and confirms previous reports from this laboratory (Walters et al., 2011a,b). Reports of the use of elicitor combinations to control plant disease are rare, although combinations of ASM and Milsana® (extract of *Reynoutria sachalinensis*) were found to control powdery mildew on cucumber (Bokshi et al., 2008).

Molecular studies on tobacco and *Arabidopsis* have shown that ASM activates the SAR pathway by mimicking the activity of SA (Gaffney et al., 1993; Friedrich et al., 1996; Lawton et al., 1996). The situation with BABA is more complex and seems to involve SA-dependent, SA-independent, and abscisic acid (ABA)-dependent mechanisms, with the relative importance of the different signaling pathways depending on the particular host-pathogen interaction (Ton et al., 2005). As indicated earlier, CJ is structurally related to JA and MeJA, although it up-regulates a unique set of genes compared to MeJA (Birkett et al., 2000; Pickett et al., 2007). Treatment of leaves one and two of barley with the combination of ASM, BABA, and CJ led to an approximately 6-fold up-regulation of *PR-1b* in leaves three and four, confirming previous reports on the effects of the elicitor combination on defense-related gene expression (Walters et al., 2011a,b). Expression of a *PR-1* gene is usually considered to be a molecular marker for SAR (van Loon et al., 2006) and therefore, these data suggest that the elicitor combination activated SAR in barley. *PR-1b* was also up-regulated by inoculation with *R. commune* (~4-fold increase in leaf 3, for example). However, the largest up-regulation of *PR-1b* was obtained when elicitor-treated plants were subsequently inoculated with *R. commune*. This suggests that the elicitor combination primed *PR-1b* gene expression in these plants. In contrast to *PR-1b*, the expression of *LOX2* exhibited a completely different trend. Treatment of barley plants with the elicitor combination resulted in a substantial down-regulation of *LOX2* (4-fold in leaf 3 and 5.5-fold in leaf 4). Inoculation with *R. commune* led to a greater down-regulation of *LOX2* in the treated leaves, while application of the elicitor combination followed by inoculation had no further effect on gene expression than the elicitor combination only. The *LOX2* gene, which is involved in the octadecanoid pathway, is auto-regulated by JA, thereby controlling a feed-forward loop in JA biosynthesis (Bell et al., 1995). Suppression of *LOX2* in transgenic *Arabidopsis* was shown to block JA biosynthesis during pathogen infection (Spoel et al., 2003), and the present data on *LOX2* in barley suggests that the elicitor combination might suppress activity of the JA pathway in barley. Concomitant activation of SAR and suppression of the JA pathway might be expected to enhance defense against biotrophic pathogens and increase susceptibility to necrotrophic pathogens (Glazebrook, 2005). *R. commune* is a hemibiotrophic pathogen, possessing an initial biotrophic phase followed by a prolonged necrotrophic phase (Walters et al., 2008). Perhaps it is no surprise therefore, that the elicitor combination reduces infection by *R. commune*. It is possible that the elicitor combination might compromise the ability of the plant to defend itself against necrotrophic pathogens and indeed, the elicitor combination was shown to provide control of powdery mildew and *R. commune*, but was associated with increased symptoms of leaf spot caused by the necrotrophic pathogen *Ramularia collo-cygni* on spring barley in the field (Walters et al., 2011b). Plant defense against chewing insects is mediated by JA signaling (Pieterse et al., 2012), as is establishment of functional arbuscular mycorrhizal (AM) symbioses (Pozo and Azcón-Aguilar, 2007). In view of the down-regulation of *LOX2* in barley treated with the elicitor combination, it would be prudent to examine the effects of treated plants on defense against herbivorous insects and on the establishment of AM symbiosis.

In all 3 years of field experiments, the elicitor combination applied on its own was either partially effective or ineffective at controlling powdery mildew and *R. commune* on the two barley cultivars. In 2009, the elicitor combination provided significant control of both diseases on both spring barley varieties, and also controlled powdery mildew on Optic in 2008. However, the elicitor treatment did not control *R. commune* on either variety in 2008 and provided no disease control in 2007. Perhaps unsurprisingly therefore, given the poor levels of disease control provided by the elicitor-only treatment, grain yield was not significantly affected, apart from 2007, when grain yield of Optic was significantly reduced compared to the untreated control. Here, the reduced grain yield probably reflected the significantly reduced GLA in the elicitor-only treatment. Infection by many pathogens, including biotrophic pathogens such as powdery mildews, results in chlorosis and reduced photosynthetic rates (e.g., Walters and McRoberts, 2007) and the failure of the elicitor-only treatment to control infection would probably have affected rates of photosynthesis. Whether the elicitor combination affects photosynthesis in barley is not known, but should be examined.

The data on disease control presented in this paper highlight two typical problems associated with the practical use of elicitors on certain crops under field conditions, namely inconsistency and poor levels of disease control (Walters and Fountaine, 2009). In contrast to the elicitor-only treatments, the performance of the elicitor plus fungicide treatment was better both in terms of disease control and consistency. The acid test for such combined treatments is whether the performance of the combination is superior to that of the fungicide treatment on its own. Unfortunately, in most cases, although the combined elicitor and fungicide treatment performed as well as the fungicide-only treatment in terms of disease control, only in one case (control of *R. commune* on Optic in 2008) did the elicitor plus fungicide treatment out-perform the fungicide-only treatment. A similar situation was found for grain yield. Here, although grain yield tended to be increased by application of the elicitor plus fungicide treatment, most of these increases were not significantly different from the fungicide-only treatment. The lack of consistency in terms of disease control shown by the elicitor combination in barley, contrasts with the situation in oilseed rape (*Brassica napus*). Here, application of the elicitor combination to winter oilseed rape provided better control of light leaf spot caused by *Pyrenopeziza brassicae*, than standard fungicide treatments (Oxley and Walters, 2012).

Over the 3 years of field experiments, the elicitor plus fungicide combinations providing most consistent disease control were treatments 13, 14, and 16. As indicated earlier, only data for treatment 13 are shown, since this treatment performed most consistently throughout the study. Treatment 13 involved application of a combination of fungicide at reduced rate plus elicitor at GS39, with no control treatments applied at earlier growth stages. It has been suggested that application of elicitors earlier in the season might reduce inoculum levels, thereby requiring less fungicide to be applied later (Walters et al., 2013). On the basis of the results obtained in the present paper, this suggestion does not appear to work for spring barley. This suggests that, at least for spring barley, protecting later stages of crop growth is important in maintaining grain yield.

In some crops, use of elicitors and fungicides (or bactericides) can be effective. For example, the use of ASM (as Actigard®) in combination with fungicides and bactericides was recommended in tomato spray programs in North Carolina, USA (Ivors and Louws, 2007). The rationale here was that the elicitor would increase plant resistance, while the fungicides and bactericides would reduce pathogen inoculum levels. On mandarins (variety Murcott), tank-mixing ASM with azoxystrobin improved the efficacy of the fungicide by more than 50% (Miles et al., 2005), although this effect was clearly variety-specific, since no extra benefit of tank-mixing the elicitor and fungicide was obtained with the mandarin variety Imperial (Miles et al., 2004).

It has been suggested that one of the reasons for the relatively poor performance of elicitors is due to the likelihood that under field conditions, plants are already induced (Walters, 2009). Indeed, Herman et al. (2007) found that in tomato under field conditions, some defense genes were already expressed prior to ASM application. Nevertheless, the expression of these genes was increased further following ASM application. In preliminary work, examination of CAD activity in leaves from the field experiment in 2007 indicated that activity of the enzyme was already high prior to elicitor treatment (Paterson and Walters, unpublished results). Although it is tempting to suggest that CAD activity was increased further following elicitor application, any increases observed were not significant. It is possible that in barley, unlike tomato, prior induction of resistance compromises the ability of the plant to respond effectively to elicitors. Indeed, this was reported for barley treated with the elicitor combination, where prior inoculation with *R. commune* compromised the ability of the plant to respond to subsequent elicitor treatment (Walters et al., 2011a). It was suggested that this might help to explain the relatively poor performance of induced resistance in the field, particularly in cereals, compared to plants grown under controlled conditions (Walters et al., 2011a).

The results presented in this paper indicate quite clearly that use of a combination of elicitors alone does not provide effective disease control in spring barley. In contrast, using the elicitor combination and fungicides, even at half-rate, can provide levels of disease control and yield increases that are equal to the best fungicide-only treatment. From a practical perspective, an elicitor plus fungicide program is only likely to be attractive to a grower if it is cost-effective i.e., it provides levels of disease control and yield increases above that achieved using the fungicide on its own. This suggests that barley growers are unlikely to find the elicitor plus fungicide treatments examined in this work an attractive proposition. This might change however, if fungicide availability is further reduced through legislation. Indeed, elicitor/fungicide combinations could be valuable in reducing fungicide use, and prolonging the useful life of certain fungicides.

### Conflict of interest statement

The authors declare that the research was conducted in the absence of any commercial or financial relationships that could be construed as a potential conflict of interest.
