# Supplementary material for: Control of foliar pathogens of spring barley using a combination of resistance elicitors
Source: Front Plant Sci. 2014 May 28;5:241. doi: 10.3389/fpls.2014.00241 (PMC4036063; doi:10.3389/fpls.2014.00241)
Supplement: Supplementary file 3 [file DataSheet3.PDF]

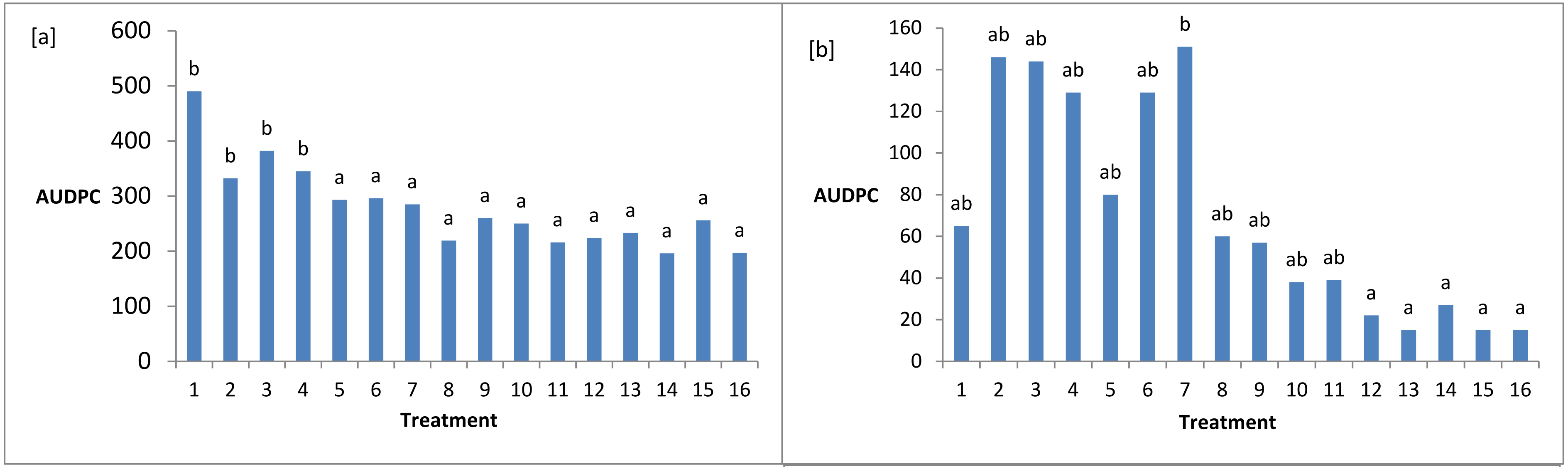

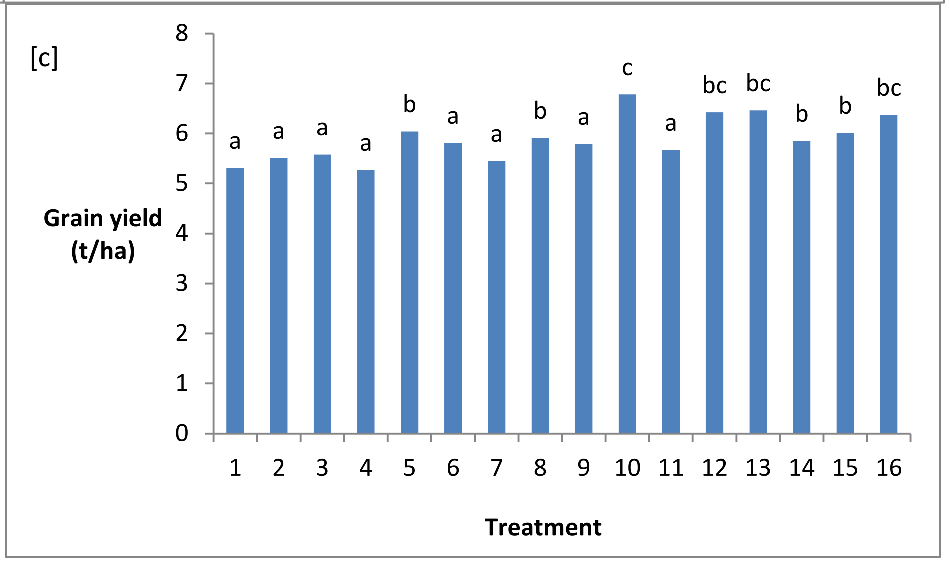

Supplemental material, Figure 3. Effects of the elicitor combination and fungicides on powdery mildew AUDPC (a), *R. secalis* AUDPC (b), and grain yield (c) in the spring barley variety Optic in 2008. Treatments T1-16 are described in Table 1. Bars with different letters are significantly different at $P < 0.05$ (Fisher's LSD).
